# Tolerability and efficacy of bimatoprost 0.01 % in patients with open-angle glaucoma or ocular hypertension evaluated in the Taiwanese clinical setting: the Asia Pacific Patterns from Early Access of Lumigan 0.01 % (APPEAL Taiwan) study

**DOI:** 10.1186/s12886-016-0338-6

**Published:** 2016-09-15

**Authors:** Ying Ying Chen, Tsing-Hong Wang, Catherine Liu, Kwou-Yeung Wu, Shin-Lin Chiu, Susan Simonyi, Da-Wen Lu

**Affiliations:** 1 Kaohsiung Veterans General Hospital, Kaohsiung City, Taiwan; 2 National Taiwan University Hospital, Taipei City, Taiwan; 3 Taipei Veterans General Hospital, Taipei City, Taiwan; 4 Kaohsiung Medical University Hospital, Kaohsiung City, Taiwan; 5 Changhua Christian Hospital, Changhua City, Taiwan; 6 Allergan Singapore Pte Ltd., Singapore, Singapore; 7 Tri-Service General Hospital, Taipei City, Taiwan

**Keywords:** Glaucoma, Normal-tension glaucoma, Ocular hypertension, Intraocular pressure, Hyperemia, Prostaglandin analog, Prostamide, Bimatoprost

## Abstract

**Background:**

In randomized, controlled trials of open-angle glaucoma (OAG) or ocular hypertension (OHT), bimatoprost 0.01 % improved tolerability while retaining the intraocular pressure (IOP)-lowering efficacy of bimatoprost 0.03 %. Given geographic/racial differences in glaucoma presentation, the APPEAL study assessed the occurrence and severity of hyperemia produced by bimatoprost 0.01 %, and its efficacy, in the Taiwanese clinical setting.

**Methods:**

In this multicenter, open-label, observational study, treatment-naïve and previously treated patients with OHT or OAG received once-daily bimatoprost 0.01 % for 12 weeks. Hyperemia (primary endpoint) was graded at baseline, week 6, and week 12 using a photonumeric scale (0, +0.5, +1, +2, +3), grouped (≤ +1, none to mild; ≥ +2, moderate to severe), and reported as unchanged from baseline, improved, or worsened. IOP assessments followed the same schedule. Supplemental efficacy analyses were conducted based on previous therapies.

**Results:**

The intent-to-treat population (*N* = 312) included treatment-naïve (13.5 %) and previously treated (86.5 %) patients; mean age was 53.3 years. At baseline, 46.3 % of previously treated patients were receiving prostaglandin analog (PGA) monotherapy. At week 12, 91.2 %, 5.9 %, and 2.9 % of treatment-naïve patients exhibited unchanged, worsened, and improved hyperemia from baseline, respectively; 77.9 %, 12.9 %, and 9.2 % of previously treated patients showed no change, worsening, and improvement, respectively. There were no statistically significant shifts in hyperemia severity in either group, or in subgroups based on previous use of any PGA, any non-PGA, latanoprost, or travoprost monotherapies. In treatment-naïve patients, mean IOP reduction from baseline (18.0 ± 3.8 mm Hg) was 3.6 mm Hg at week 12 (*P* < 0.0001); 83.3 % had baseline IOP ≤ 21 mm Hg. In previously treated patients, mean additional IOP reduction from baseline (17.8 ± 3.9 mm Hg) was 2.6 mm Hg (*P* < 0.0001); similar results were observed in patient subgroups based on previous therapies.

**Conclusions:**

In the Taiwanese clinical setting, bimatoprost 0.01 % provided significant IOP lowering in treatment-naïve patients (regardless of baseline IOP) and previously treated patients (even those with relatively low IOP on other therapies), while causing no significant changes in hyperemia from baseline.

**Trial registration:**

Clinicaltrials.gov NCT01814761. Registered 18 March 2013.

## Background

Glaucoma is second only to cataract as a leading cause of blindness worldwide [1, 2], and because the burden increases substantially with advanced disease [3–5], early detection and treatment is important. In addition, the characteristics and prevalence of glaucoma have been shown to vary with geography and race [6–11], emphasizing the need for clinical studies and management programs adapted to local populations [12]. For example, open-angle glaucoma (OAG) characterized by intraocular pressure (IOP) ≤ 21 mm Hg (i.e., normal-tension glaucoma [NTG]) is much more common in patients in Asian countries than Western ones [13]. A Korean study of adults over 50 years of age indeed showed that NTG accounted for 94.4 % of all OAG cases [14]. Similarly, another Korean study showed that 75.3 % of patients with OAG had baseline IOP ≤ 21 mm Hg [15]. However, whether OAG is associated with elevated or normal IOP, treatment options remain limited because the underlying mechanisms have yet to be elucidated. Current management of OAG relies on topical IOP-lowering agents [16, 17], and prostaglandin analogs (PGAs)/prostamides are often preferred as first-line therapy owing to their efficacy, safety, and convenience of use (once daily) [17–19]. In Taiwan, however, the National Health Insurance Administration reserves them for use as second-line therapy [20, 21].

The prostamide bimatoprost 0.03 % (Lumigan® 0.03 %; Allergan plc, Irvine, CA, USA) is an effective topical treatment with superior IOP-lowering effects (compared with other PGA monotherapies) and a favorable tolerability profile with long-term use [22–25]. Importantly, clinical studies have also demonstrated its efficacy and tolerability in patients with NTG [26–30]. Nonetheless, some patients experience adverse events (AEs) when treated with bimatoprost 0.03 %, the most common being conjunctival hyperemia. Considering that AEs experienced chronically can lead to nonadherence to therapy [31, 32] and impact disease progression [33, 34], bimatoprost 0.01 % (Lumigan® 0.01 %; Allergan plc, Irvine, CA, USA) [35] was developed and shown to improve tolerability while retaining the IOP-lowering efficacy of bimatoprost 0.03 % in clinical studies of patients with elevated IOP due to OAG or ocular hypertension (OHT) [36–39].

Given the regional differences in glaucoma presentation and treatment guidelines mentioned above [40], the Asia Pacific Patterns from Early Access of Lumigan 0.01 % study in Taiwan (APPEAL Taiwan) evaluated the occurrence and severity of hyperemia produced by bimatoprost 0.01 % monotherapy, as well as its IOP-lowering effects, in patients with OHT or OAG, including NTG, seen in the Taiwanese clinical practice setting.

## Methods

### Study design

This 12-week, open-label, noncomparative, observational study of bimatoprost 0.01 % in consecutive patients with OAG (including NTG) or OHT (ClinicalTrials.gov identifier: NCT01814761, registered on March 18, 2013) was conducted between May 2013 and August 2014, in accordance with the Guidelines for Good Clinical Practice and all applicable Taiwanese laws.

### Study population

Eligible patients were at least 20 years of age, treatment-naïve or previously treated, and had been diagnosed before screening with OHT or OAG (including NTG), according to standard of care in the treating physician’s practice. OAG was defined as an eye with glaucomatous optic nerve head change and corresponding glaucomatous visual field defects, and the decision to prescribe topical bimatoprost 0.01 % was made by the treating physician prior to, and without consideration of study participation (per standard of care). Key exclusion criteria included a history of bimatoprost 0.01 % use; contraindications to bimatoprost 0.01 % use; hypersensitivity to any PGA or component of the study medication; concomitant use of topical, periorbital, intravitreal, or systemic steroid within 3 months of study initiation, or anticipated use during the study; and presence of any other abnormal ocular condition or symptom preventing study participation.

### Study treatment

Bimatoprost 0.01 % was provided by Allergan Singapore Pte Ltd. At the baseline visit, patients were instructed to instill 1 drop of medication in the study eye each evening (at approximately 8 pm). If both eyes were eligible for inclusion, both were treated, but the eye with the higher baseline IOP was included in the analysis. If both eyes had identical baseline IOP, the right eye was designated as study eye. There was no washout of previous IOP-lowering medications prior to initiation of study treatment.

### Outcomes and analyses

All outcomes were measured at approximately the same time of day at baseline, and weeks 6 and 12. The primary outcome variables were the occurrence and severity of ocular hyperemia at week 12, assessed and graded using a standard photonumeric bulbar conjunctival hyperemia grading scale: 0 (none; normal); +0.5 (trace; trace flush, reddish pink); +1 (mild; mild flush, reddish color); +2 (moderate; bright red color); and +3 (severe; deep, bright diffuse redness). Hyperemia grading was then collapsed into 2 categories: none to mild (i.e., 0 to +1) and moderate to severe (i.e., +2 and +3), as described by other groups [15, 38, 41, 42], and the shift in hyperemia severity/grading from baseline at weeks 6 and 12 was reported as improved, unchanged, or worsened [38].

The secondary outcome variables included the change in IOP from baseline (based on Goldmann applanation tonometry performed per standard of care in the treating physician’s practice) and response rates (i.e., percentage change in IOP from baseline) at 6 and 12 weeks in treatment-naïve patients and patients previously treated with monotherapy or combination therapy (i.e., switched). Safety assessments included biomicroscopy, visual acuity, ocular AEs, and the number of discontinuations due to AEs. For each discontinuation, every effort was made to contact the patient and document the outcomes.

All patients who received at least 1 dose of study medication were included in the intent-to-treat and safety populations. All individual hyperemia scores recorded for each grade (i.e., 0, +0.5, +1, +2, +3) were reported as frequency counts and percentages for all study visits, and the change from baseline was reported at weeks 6 and 12. The treatment effect was analyzed using a 2-sided McNemar test. Change and percentage change in IOP from baseline at weeks 6 and 12 were analyzed using the 2-sided Student paired *t* test. No statistical analyses were conducted comparing treatment-naïve and previously treated patients. Owing to the exploratory nature of the study, no sample size calculation was carried out.

Supplemental analyses of the occurrence and severity of hyperemia produced by bimatoprost 0.01 %, as well as its IOP-lowering effects, were conducted for subgroups of patients who at baseline were receiving PGA or non-PGA monotherapy, and latanoprost or travoprost monotherapy.

## Results

**Fig. 1:**
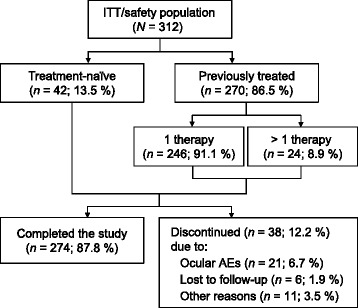
Patient disposition. *AEs* adverse events, *ITT* intent-to-treat

Eleven centers participated in the study. The intent-to-treat/safety population (*N* = 312) consisted of treatment-naïve patients and previously treated patients (Fig. 1). Among the latter subgroup, 91.1 % had been prescribed only 1 IOP-lowering therapy (monotherapy or fixed-combination therapy of any kind) at the time of enrollment. Most patients completed the study, and discontinuations were due to ocular AEs, lost to follow-up, or other non-AE-related reasons (Fig. 1).

Overall, 90.4 % of patients had a diagnosis of OAG. Baseline characteristics were similar between treatment-naïve and previously treated patients, except for the diagnosis and medical comorbidities (Table 1). Among previously treated patients, 83.0 % (*n* = 224) were receiving monotherapy at baseline (Table 1); 93.0 % were switched to bimatoprost 0.01 % monotherapy because of intolerance or insufficient IOP-lowering with previous therapy.

**Table 1:** Patient demographics and characteristics at baseline

Characteristic	Treatment-naïve (*n* = 42)	Previously treated (*n* = 270)	All (*N* = 312)
Age, mean (SD), years	52.5 (13.7)	53.4 (14.5)	53.3 (14.3)
Gender, *n* (%)			
Male	22 (52.4)	156 (57.8)	178 (57.1)
Female	20 (47.6)	114 (42.2)	134 (42.9)
Diagnosis, *n* (%)			
OHT	9 (21.4)	21 (7.8)	30 (9.6)
OAG	33 (78.6)	249 (92.2)	282 (90.4)
Study eye, *n* (%)			
Right	27 (64.3)	171 (63.3)	198 (63.5)
Left	15 (35.7)	99 (36.7)	114 (36.5)
IOP, mean (SD), mm Hg	18.0 (3.8)	17.8 (3.9)	17.9 (3.9)
Patients previously treated^a^, *n* (%)	NA		
PGA monotherapy		125 (46.3)	125 (40.1)
Non-PGA monotherapy		99 (36.7)	99 (31.7)
PGA fixed-combination therapy		5 (1.9)	5 (1.6)
Non-PGA fixed-combination therapy		17 (6.3)	17 (5.4)
> 1 prior therapies		24 (8.9)	24 (7.7)
Patients with ≥ 1 medical comorbidities^b^, *n* (%)	14 (33.3)	160 (59.3)	174 (55.8)

^a^Medications used at baseline included betaxolol, bimatoprost 0.03 %, brimonidine, brimonidine/timolol, brinzolamide, brinzolamide/timolol, carteolol, dorzolamide, dorzolamide/timolol, latanoprost, latanoprost/timolol, timolol, and travoprost

^b^Medical comorbidities included asthma, diabetes, hypertension, cardiovascular disease, pulmonary disease, periorbital changes due to previous PGA therapy, and others

*IOP* intraocular pressure, *NA* not applicable, *OAG* open-angle glaucoma, *OHT* ocular hypertension, *PGA* prostaglandin analog, *SD* standard deviation

**Fig. 2:**
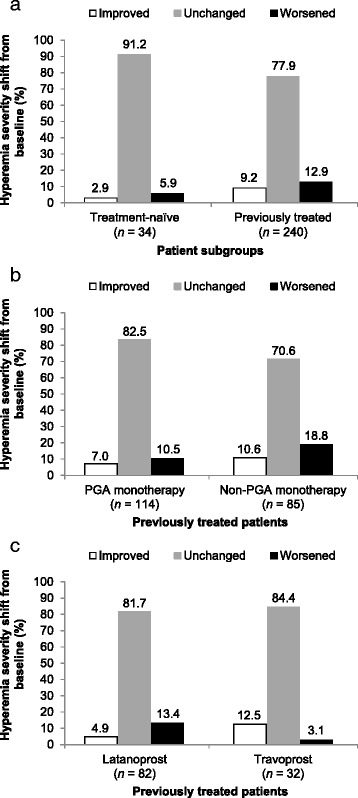
Shift in hyperemia severity grading from baseline at week 12 in (**a**) treatment-naïve and previously treated patients (*P* ≥ 0.2717 in both groups, compared with baseline), (**b**) patient subgroups previously treated with prostaglandin analog (PGA) or non-PGA monotherapy (*P* ≥ 0.2295 in both groups, compared with baseline), and (**c**) patient subgroups previously treated with latanoprost or travoprost monotherapy (*P* ≥ 0.1185 in both groups, compared with baseline)

At week 12, 29 (85.3 %) treatment-naïve patients with available data had no hyperemia, compared with 36 (85.8 %) at baseline (Table 2). No severe cases were reported in this group of patients at any visit (Table 2). In the previously treated group, 170 (70.8 %) patients had no hyperemia at week 12, compared with 201 (74.4 %) at baseline (Table 2; Fig. 2a). There were no statistically significant shifts in hyperemia severity from baseline in either group (Fig. 2a) or in subgroups based on previous use of non-PGA versus PGA monotherapy (Fig. 2b), and latanoprost versus travoprost monotherapy (Fig. 2c). Similarly, there were no statistically significant shifts in hyperemia severity from baseline (*P* = 1.0000) in patients previously treated with > 1 therapy (not shown).

**Table 2:** Occurrence of ocular hyperemia by severity grade

	Treatment-naïve (*n* = 42)		Previously treated (*n* = 270)	
Assessment visit	Baseline	Week 12	Baseline	Week 12
Missing data, *n* (%)	0	8 (19.0)	0	30 (11.1)
Hyperemia grading, *n* (%)				
0 (none, normal)	13 (31.0)	7 (20.6)	39 (14.4)	36 (15.0)
+0.5 (trace)	12 (28.6)	10 (29.4)	70 (25.9)	50 (20.8)
+1 (mild)	11 (26.2)	12 (35.3)	92 (34.1)	84 (35.0)
+2 (moderate)	6 (14.3)	5 (14.7)	57 (21.1)	59 (24.6)
+3 (severe)	0	0	12 (4.4)	11 (4.6)

**Fig. 3:**
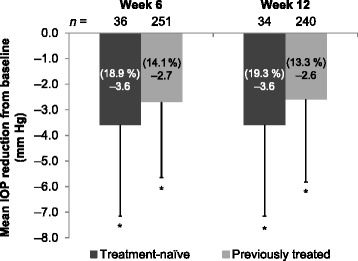
Mean intraocular pressure (IOP) reduction at weeks 6 and 12 in treatment-naïve and previously treated patients. *P < 0.0001, compared with baseline

In treatment-naïve patients, mean baseline IOP ± SD was 18.0 ± 3.8 mm Hg. A statistically significant reduction in mean IOP from baseline of approximately 20 % was observed at weeks 6 and 12 (*P* < 0.0001; Fig. 3). In the subgroup of patients who had a baseline IOP ≤ 21 mm Hg (mean ± SD, 16.6 ± 2.3 mm Hg; *n* = 35; 83.3 %), the mean IOP reduction from baseline was 2.8 mm Hg (*P* < 0.0001 at both timepoints) at both weeks 6 (16.2 %) and 12 (16.6 %). In the subgroup who had a baseline IOP > 21 mm Hg (mean ± SD, 24.9 ± 2.0 mm Hg; *n* = 7; 16.7 %), the mean IOP reduction from baseline reached 10.5 ± 4.2 mm Hg (*P* = 0.0154) at week 6 (41.0 %) and 10.0 ± 4.1 mm Hg (*P* = 0.0163) at week 12 (39.0 %).

**Fig. 4:**
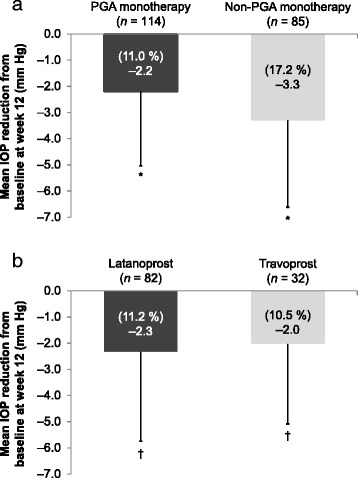
Mean intraocular pressure (IOP) reduction at week 12 in (**a**) patient subgroups previously treated with prostaglandin analog (PGA) or non-PGA monotherapy, and (**b**) patient subgroups previously treated with latanoprost or travoprost monotherapy. *P < 0.0001, compared with baseline; ^†^ *P* < 0.0002, compared with baseline

In previously treated patients, mean IOP ± SD at baseline was 17.8 ± 3.9 mm Hg, and the mean additional IOP reduction from baseline (> 13 %) was also statistically significant at both post-baseline visits despite the high proportion of patients previously treated with PGA monotherapy (*P* < 0.0001; Fig. 3; Table 1). Similar results were observed in the patient subgroups previously treated with PGA versus non-PGA monotherapy (Fig. 4a), as well as those previously treated with latanoprost versus travoprost monotherapy (Fig. 4b). In patients who received > 1 therapy before switching to bimatoprost 0.01 %, mean IOP ± SD at baseline was 18.4 ± 3.8 mm Hg, and the mean additional IOP reduction from baseline was also statistically significant at weeks 6 (3.3 mm Hg; *P* = 0.0001) and 12 (2.4 mm Hg; *P* = 0.0093).

Overall, 26.2 % and 13.7 % of treatment-naïve and previously treated patients reported treatment-related AEs, respectively (Table 3); all were ocular in nature, none were serious, and conjunctival hyperemia was the most frequent in both groups (Table 3). Consistent with these results, more patients discontinued treatment owing to AEs in the treatment-naïve and previously treated groups (Table 3).

**Table 3:** Summary of patients with adverse events^a^

	Treatment-naïve (*n* = 42)	Previously treated (*n* = 270)	All (*N* = 312)
All adverse events, *n* (%)	18 (42.9)	70 (25.9)	88 (28.2)
Treatment-related, *n* (%)	11 (26.2)	37 (13.7)	48 (15.4)
Ocular^a^	11 (26.2)	37 (13.7)	48 (15.4)
Ocular hyperemia	6 (14.3)	12 (4.4)	18 (5.8)
Dark circles under the eyes	0	6 (2.2)	6 (1.9)
Eye pruritus	1 (2.4)	4 (1.5)	5 (1.6)
Decreased lacrimation	1 (2.4)	4 (1.5)	5 (1.6)
Eye allergy	1 (2.4)	2 (0.7)	3 (1.0)
Ocular discomfort	1 (2.4)	1 (0.4)	2 (0.6)
Eye pain	1 (2.4)	0	1 (0.3)
Serious	0	0	0
Leading to discontinuation, *n* (%)	6 (14.3)	15 (5.6)	21 (6.7)

^a^Reported by > 2 % of patients

## Discussion

The APPEAL Taiwan study was designed to assess the tolerability and efficacy of bimatoprost 0.01 % in patients with OHT or OAG (including NTG) evaluated in typical clinical practice settings. The results demonstrate that when administered once daily over 12 weeks, bimatoprost 0.01 % caused no significant shift in hyperemia severity from baseline in treatment-naïve and previously treated patients, but produced statistically significant IOP lowering from baseline in both groups.

At study end, hyperemia severity remained unchanged or improved in ≥ 87 % of patients in both groups. Moreover, mean IOP reduction from baseline reached 19 % in treatment-naïve patients, despite a low baseline mean IOP (i.e., 18.0 ± 3.8 mm Hg). Although the subgroup of patients with baseline IOP > 21 mm Hg and available data on hyperemia at week 12 was small (*n* = 4), mean IOP reduction from baseline reached 39 %, consistent with data from other studies of bimatoprost 0.01 % in treatment-naïve patients with baseline IOP > 21 mm Hg [15, 38, 39, 43]. Similarly, bimatoprost 0.01 % provided an additional 13 % reduction in mean IOP in previously treated patients, compared with baseline, despite the heterogeneity of this subgroup in terms of previous treatment.

In the supplemental analyses based on previous treatment, statistically significant reductions in mean IOP from baseline were observed in patient subgroups previously treated with non-PGA or PGA monotherapy, latanoprost or travoprost monotherapy, or > 1 therapy before switching to bimatoprost 0.01 %, but no significant shifts in hyperemia severity from baseline were observed in any of those subgroups.

Overall, our data indicate that treatment with bimatoprost 0.01 % can significantly lower IOP in treatment-naïve patients who have a low baseline IOP, as well as patients who have achieved some degree of IOP lowering with other therapies. These findings are consistent with those of the multicenter, open-label, observational, APPEAL Korea study of treatment-naïve [15] and previously treated [42] patients with OAG (including NTG) or OHT evaluated in the Korean clinical setting. The IOP-lowering efficacy and tolerability of bimatoprost 0.01 % observed herein also are consistent with the results of a 12-month, multicenter, randomized, double-masked, controlled clinical trial of bimatoprost 0.01 % in treatment-naïve and previously treated patients with elevated IOP due to glaucoma or OHT [37]. Similarly, our results are consistent with those of the 12-week, open-label, multicenter, observational, Canadian CLEAR study of treatment-naïve patients with OAG or OHT and elevated IOP who were monitored in the clinical setting [38].

The overall percentage of patients with ocular AEs leading to discontinuation (6.7 %) recorded herein is consistent with that reported in other studies (i.e., ≤ 5.4 %) [15, 37–39, 44].

As age is a primary risk factor for OAG and the population is aging across the globe, more patients are expected to require monitoring and therapy in the near future [1], making OAG an important clinical issue. In this regard, it is noteworthy that the study population evaluated herein had a relatively young mean age (53.3 years), compared with that in other studies of bimatoprost 0.01 % (mean range, 61.1–68.2 years) [37–39, 44]. In fact, mean age was even lower than the 58.0 and 59.5 years reported in the APPEAL Korea study of treatment-naïve [15] and previously treated patients [42], respectively, which included patients with NTG. Whether this difference is indicative of early onset or early detection is unknown at this time and requires further investigation.

Potential limitations of the study include the open-label design, lack of comparator, relatively short duration, and a possible Hawthorne effect in patients who switched to bimatoprost 0.01 % from a previous treatment [41], as well as the fact that inter-observer differences could have existed in the hyperemia grading despite use of a standard photonumeric bulbar conjunctival hyperemia grading scale. The absence of a washout period prior to initiation of study treatment should also be considered, although it was deliberately set to reflect typical clinical practice settings. It is also noteworthy that all outcome variables were assessed at 12 weeks, a timepoint at which no residual carry-over effects from previous treatments are expected.

## Conclusions

This study of patients with OAG (including NTG) or OHT in the Taiwanese clinical setting showed that bimatoprost 0.01 % provides significant IOP lowering in treatment-naïve patients (regardless of baseline IOP), as well as previously treated patients (even those with relatively low IOP on other therapies), while causing no significant changes in hyperemia from baseline.

## Abbreviations

AEs: Adverse events
IOP: Intraocular pressure
NTG: Normal-tension glaucoma
OAG: Open-angle glaucoma
OHT: Ocular hypertension
PGA: Prostaglandin analog
SD: Standard deviation
